# The Combined Elevation Test (CET) in Adolescent School Children: A Pilot Study

**DOI:** 10.3390/sports6030064

**Published:** 2018-07-20

**Authors:** James Furness, Ben Schram, Darren Corea, Zachary Turner, Hannah Cairns

**Affiliations:** 1 Water Based Research Unit, Bond Institute of Health & Sport, Bond University, Gold Coast, QLD 4226, Australia; jfurness@bond.edu.au; 2 Faculty of Health Sciences and Medicine, Bond University, Gold Coast, QLD 4226, Australia; darren.corea@student.bond.edu.au (D.C.); zachary.turner@student.bond.edu.au (Z.T.); Hannah.cairns@student.bond.edu.au (H.C.)

**Keywords:** combined elevation test, musculoskeletal screening test, normative data, swimming, adolescent

## Abstract

The Combined Elevation Test (CET) is a musculoskeletal screening technique (MST) that replicates the streamline position in swimming and is commonly used in various sports. Although CET is widely used, no normative data exist within an adolescent population. Therefore, the purpose of this study was to develop a large data set for the CET within an adolescent population and to evaluate the influence of various demographic and anthropometric variables. Data were collected for 416 participants aged between 8 and 18 years old. Age and arm span showed a significant correlation with CET scores (arm span r*_s_* (105) = 0.478, *p* = 0.000; age r*_s_* (416) = 0.238, *p* = 0.000). Regression analysis further quantified the influence of arm span and age on CET scores, accounting for 23.1% and 5.3% of variability respectively. These results can be used as a reference point for clinicians and coaches who are using the CET within their assessments.

## 1. Introduction

Sport-specific musculoskeletal screening techniques (MSTs) are used in the identification of intrinsic risk factors for injury [1], determining whether a patient can achieve a minimum standard for participation in a particular sport and to assist in appropriate goal setting. Poor performance on an MST may indicate movement dysfunction, restriction or asymmetry and may predispose the athlete to injury or identify incomplete recovery from a previous injury [2].

MST selection is based on both specificity to a particular sport and the location of common injuries within that sport. In swimming, in particular, shoulder pain has been shown to be prevalent in 40–91% of participants [3]. Sein, et al. [4] revealed higher rates (91%) of reported shoulder pain in the younger swimming populations (13–25 years). The high frequency of shoulder injury, especially in younger swimmers, provides the rationale for the use MSTs to screen for injury risk.

The Combined Elevation Test (CET) is a MST originally developed by Blanch [5] that involves a synchronized movement of thoracic extension, glenohumeral joint (GHJ) flexion, scapula retraction and upward rotation [2]. With relevance to the sport of swimming, these movements replicate a streamline position required to reduce drag forces [5]. Maintaining a streamline position requires both strength and flexibility of a swimmer [5]. Flexibility is required at both the shoulder and thoracic spine to successfully perform the CET.

The comparative flexibility of one area to another part of the body (relative flexibility) along the kinetic chain is highly relevant to swimming. For example, when there is a loss of range of motion (ROM) in one joint along the kinetic chain, other joints will compensate to maintain the desired position. With relevance to freestyle swimming, high humeral elevation is needed to assist with propulsive forces; if the GHJ cannot provide adequate movement the scapula-thoracic or spine will compensate to provide this ROM. Given the importance of flexibility at the shoulder and thoracic spine in the sport of swimming, the CET is well-placed as a MST. 

While there appear to be adequate MSTs to assess thoracic rotation [6,7], there remains a paucity of MST’s which aim to assess thoracic extension [8]. However, the CET has been widely used in sports such as cricket, rugby union, triathlon and surf lifesaving [8,9,10]. Ironically, despite the CET being designed for swimming, there appears to be limited research using the CET specifically within this cohort. Despite this, shoulder flexibility (GHJ-specific) has been widely studied in swimming cohorts with mixed results in terms of whether increased flexibility leads to higher rates of injury [5,11,12]. Blanch [5] suggests that perhaps the research needs to shift away from having a specific cut-off value to discriminate between uninjured and injured towards a range of values to determine the ‘optimal range of ROM’. Here swimmers who have greater or less than the optimal range of ROM are thought to be more likely to be injured. To determine an optimal range of ROM, large injury surveillance prospective studies are needed.

To date, the studies utilizing the CET have used relatively small sample sizes [2,8,9,10] (less than 100) and minimal research has investigated the influence of anthropometric or demographic characteristics on CET scores. Carter, Marshall and Abbott [10] performed one of the few studies which analyzed the effect of demographics on CET scores, and found that increased training volume decreased CET scores. If a clinician was to implement the CET on an uninjured patient there is very little information on what factors need to be considered when interpreting scores (i.e., how does age and arm span influence scores). Ideally, establishing a normative data set with a large enough sample size to precisely characterize a population to allow for appropriate interpretation and generalization of results is needed [13]. Furthermore, research using longitudinal injury surveillance methodologies would then determine an optimal ROM required when using the CET as a MST. 

While normative data have been established for various MSTs [14,15] no normative data exist for the CET. Despite the establishment of a robust normative data set with longitudinal tracking of injuries being a logical future direction, it was outside the scope of this research project. Consequently, the aim of this study was to present a large data set for an adolescent cohort and evaluate the influence of demographic and anthropometric variables on CET scores with the intent that clinicians and coaches will be able to utilize the current data as a baseline to compare with the results of the athlete they are testing.

## 2. Materials and Methods

### 2.1. Participants

An observational study was designed in which data collection took place between January and March 2017 at local secondary schools. This study was approved by the Queensland Department of Education and Training (550/27/1668) and the Bond University Human Research Ethics Committee (0000015415). A total of 416 participants were included in this study, with ages ranging from 8 to 18 years. All participants were provided with a verbal explanation of expectations and relevant risks associated with participation prior to commencement. All participants were required to have a consent form signed by an adult or guardian. Key demographic information collected included: age, arm span, sport involvement, injury history, and current training volume. Each participant was required to disclose any ongoing or past injuries which may affect their ability to complete the test. Participants who had an existing shoulder injury or upper body injury in the 3 months prior to testing were excluded from the study.

### 2.2. Equipment

**Figure 1:**
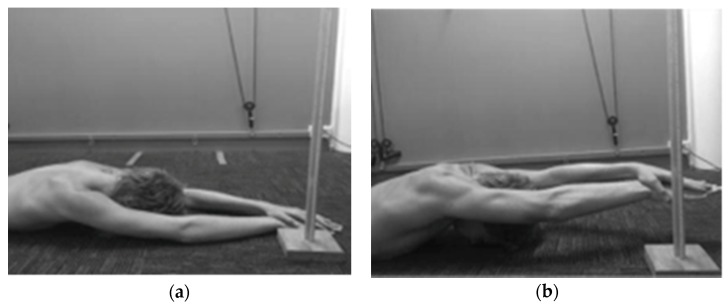
(**a**) Starting position for the Combined Elevation Test (CET); (**b**) Finishing Position for the CET.

**Figure 2:**
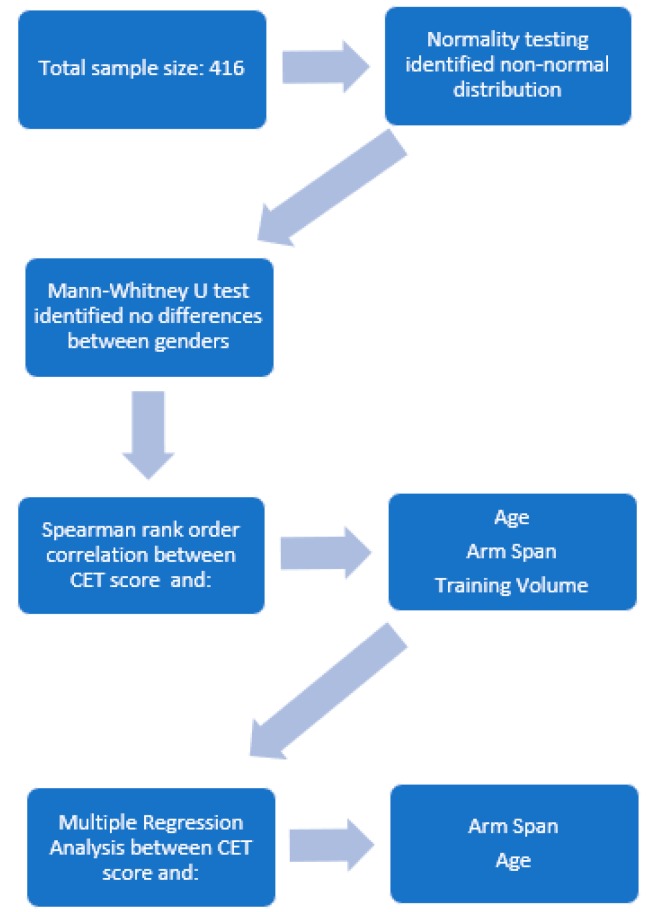
The statistical process used in this study.

A measuring stand with a 1 mm incremental scale running on a single side of the stand was used to measure combined elevation; demonstrated in Figure 1 and Figure 2. The height of the base of the measuring stand was added to each measure taken.

### 2.3. Testing Procedure

Measurements were taken by three second-year post-graduate physiotherapy students. The students received formal training on the CET procedures prior to commencement of the study from a senior physiotherapist with over 10 years of clinical experience. A small pilot study was conducted to ensure reliability of the current testing protocol; these data were not included in the final results presented. Previous research has revealed good intra- and inter-rater reliability when using the CET (ICC 0.89 and 0.97, respectively) [2].

Testing procedures were based on previously established methodology [8,10] in which participants were required to lie prone on the floor and assume a streamline swimming position. They were then asked to place their forehead, chest, hips, knees and feet on the floor (Figure 1). Forehead contact with the floor was used opposed to chin contact as a study by Allen, Phillips and McCaig [8] found that shoulder range of motion was limited in the chin position as opposed to the forehead position.

Instruction was then provided to assume a posture with their left hand on top of their right: elbows, wrists and palms straight and fully extended. Participants were required to hold a neutral wrist position, determined by the position of the metacarpals in relation to the ulna. Measurements were only taken when the patient’s metacarpals were aligned with the ulna in the sagittal plane. Participants were then instructed to maximally raise their arms away from the floor, while their forehead, chest, pelvis, and feet maintained contact with the floor (Figure 1). The perpendicular distance between the base of the metacarpo-phalangeal joint (MCPJ) of the third finger and the floor was then measured and recorded for analysis.

Three sub-maximal attempts were performed as a warm up to familiarize the participants with the movement required. Following the warm up, each participant performed three maximal efforts of the CET. A rest period of 10 s between each performance was given and measurements were then collected and entered into a spreadsheet for further analysis.

### 2.4. Data Analysis

Data analysis was performed using SPSS version 23.0. Descriptive statistics including means, standard deviations and ranges were calculated to establish a normative data set. To test for normality of the data set, a Shapiro-Wilk test (*p* > 0.05) (Shapiro and Wilk, 1965) was conducted. A Mann-Whitney U test was performed to determine differences in CET scores between males and females. Spearman’s rank-order correlations were calculated to determine the associations between span, average training volume and CET. To assess the influence of age and arm span on CET scores a multiple regression analysis was performed. Statistical significance was set at *p* < 0.05. Due to the diverse range of sporting involvement (17 different sports) data were not stratified based on individual sports. Figure 2 displays the statistical process with the current data set.

## 3. Results

### 3.1. Reliability Analysis

A subset of 23 participants was used to determine the intra-rater, within-session reliability of the testing procedure. The ICC_3,1_ values and 95% confidence intervals were 0.991 and 0.983–0.996 respectively. The standard error of measurement (SEM) was 1.46 cm which was calculated based on the formula SEM = $\sqrt{\mathrm{WMS}}$, where WMS is the square error from the ANOVA [16].

### 3.2. Participant Demographics

In total, 416 participants were assessed in this study, with more males (56%), than females (44%). Participant characteristics of both age and gender are shown in Table 1. Table 1 provides a breakdown of the number of participants within each age group for both females and males. Age groups ranged from 8 to 18 years old with the greatest number of participants being between 12 and 16 years of age (89.4%, Table 1). The overall average CET score for males versus females was 19.38 ± 7.53 cm and 20.09 ± 7.89 cm respectively.

### 3.3. Comparative Analysis for Males vs. Females

A Mann-Whitney U test was used to determine differences in CET score between males and females. CET scores for males (mean = 19.38, SD = 7.53) and females (mean = 20.09, SD = 7.89) were not statistically significantly different, *U* = 20507.500, z = −0.647, *p* = 0.518. Given this finding, for all subsequent analyses, male and female results were pooled.

### 3.4. Correlations between CET Scores and Key Variables: Arm Span, Age, and Average Training Volume

Arm span data were collected on 105 participants. Spearman’s rank-order correlation tests were used to assess the relationship between arm span (n = 105), age (n = 416) and CET score within participants. Age, and arm span showed significant correlations with CET scores, with arm span showing a moderate positive correlation, r*_s_* (105) = 0.478, *p* = 0.000, and age having a low correlation, r*_s_* (416) = 0.238, *p* = 0.000. There was a non-significant correlation between average training volume per year and CET scores collected, r*_s_* (205) = 0.076, *p* = 0.277.

### 3.5. Multiple Regression Analysis: Influence of Arm Span and Age on CET Scores

**Figure 3:**
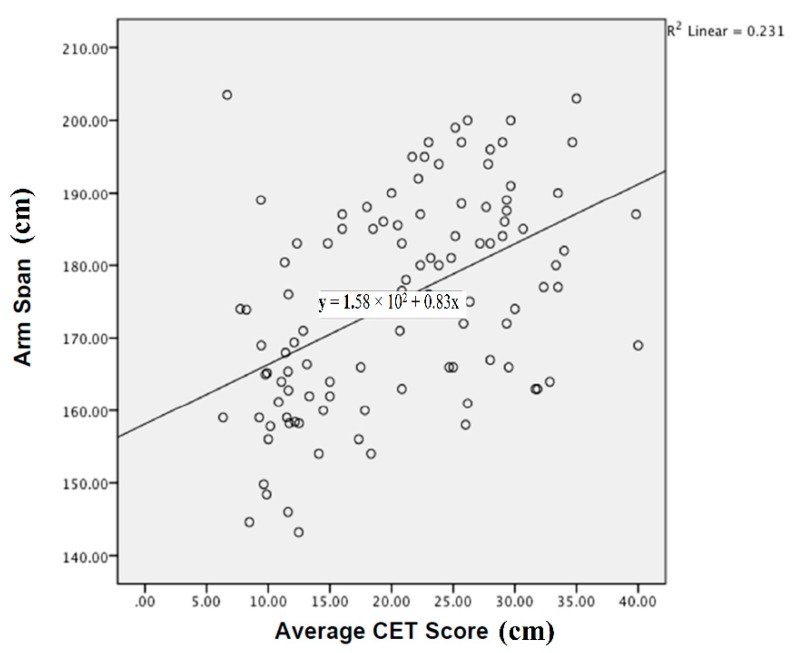
Scatterplot depicting the linear relationship between arm span (cm) and CET scores (cm).

A multiple regression analysis was used to assess the influence of arm span and age on CET scores. The multiple regression model which included arm span and age statistically significantly predicted CET scores, F(2, 102) = 20.252, *p* < 0.001, R^2^ = 0.284. Arm span, without including age, also significantly predicted CET scores, F(103, 1) = 30.996, *p* < 0.001, R^2^ = 0.231. Figure 3 presents this linear relationship graphically, with increases in arm span being associated with increases in CET scores. Therefore, 28.4% of the variation in CET scores is predicted by arm span and age (23.1% and 5.3% respectively).

### 3.6. Standardized CET Scores Based on Age and Arm Span

Given the results from the multiple regression analysis, average CET score values were stratified based on age groups and arm span groups (Table 2). Age was separated into three groups with three corresponding arm span sub-groups. The highest average CET score was recorded for the 16–18 year old group within the 190–205 cm arm span sub group (30.3 ± 4.4). The lowest scores were recorded for the 10–12 year old group within the 140–154 cm arm span sub group 12.5 ± 3.5.

## 4. Discussion

The aim of this study was to present a large CET data set for an adolescent cohort and evaluate the influence of demographic and anthropometric variables on CET scores. The intention was to provide data for both clinicians and coaches who could use it for a baseline comparison in the athlete they are testing. To the authors knowledge this is the first study to present data of this sample size and to evaluate the influence anthropometric variables have on CET scores. The key findings of this study were that both age and arm span correlated with CET scores.

The results of this study found no significant differences between males and females which aligns with research findings from Allen et al. [8] who also looked at gender differences in CET scores. This finding may be unique to the CET as previous research has illustrated gender differences in mobility favoring females [17,18]. Rikken-Bultman, Wellink and van Dongen [17] who identified increased mobility in Dutch female school children also found that the non-dominant body side was significantly more mobile than the dominant side. This may provide some rationale for the findings of the current study as the CET is a test of bilateral range of motion. This may influence CET results as outcomes would reflect the participants’ least mobile side.

The results of the current study revealed correlations between age, arm span and CET scores with increases in both age and arm span being associated with increases in CET scores. Regression analysis further quantified the influence of arm span and age in CET scores accounting for 23% and 5% of variability respectively.

One would assume that as maturity increases CET scores would decrease, as reductions in flexibility with an increase in maturity have previously been shown [17]. The authors concede two possible explanations for the findings of the current study; (1) as maturity increases so does arm span [19]; and (2) as age increases in the first two to three decades of life so does muscle mass and strength [20]. While the latter was not assessed in the current study, it is a well-known physical adaption associated with maturation [20,21]. Therefore, it is hypothesized that increases in strength and muscle mass allow for greater arm clearance during the CET. It could also be suggested that the CET is not only a measure of flexibility within the shoulder and thorax region but also muscular strength. As neither muscle mass nor strength were assessed within this current study, future research should include these variables. Since other variables may influence CET scores caution should be applied when interpreting the results of the current study. Further research is needed to determine the influence of other variables including strength, pain, and contribution from individual regions (shoulder, thoracic spine).

Given the influence that age and arm span have on CET scores the authors were able to categorize CET scores based on both variables. To the authors knowledge this is the first study to present this information. Clinicians and coaches can utilize the current data as a baseline to compare with the results of the athlete they are testing. It needs to be noted that a poor CET score of an individual within their respective category in isolation would not be able to indicate any cause for this outcome. Discrepancies would require further clinical investigation as the CET does not differentiate between joints that may be contributing towards a low CET score [8]. It also needs to be noted that the CET does not evaluate the passive restraints of the shoulder complex. There is a spectrum of shoulder joint mobility which is due to joint capsule and ligamentous laxity [22]. A poor result in the CET would require further ligamentous assessment by the clinician.

This is the largest study to date (n = 416) specific to the CET which presented a relatively gender matched data set for an adolescent age range. Wider age ranges (those older than 18 years) should also be investigated to allow for greater generalization of results and further establish the effects of age on CET scores. Furthermore, future research needs to evaluate the effectiveness of measuring shoulder joint range of motion with a goniometer, as other studies have indicated that performance in the CET is strongly related to range of GHJ flexion [8]. A major limitation of the current study is the inability to determine an optimal range of ROM. This optimal ROM would be associated with lower rates of impingement and hypermobility. This would have been possible if shoulder pain or injury data had been collected or clinical tests such as assessment of ligamentous laxity conducted. Furthermore, in an ideal situation prospective longitudinal injury surveillance methodology would be used. Unfortunately, conducting this type of study was not feasible. Given the inability to determine an optimal ROM for the CET, this current study provides a coach or clinician with baseline data stratified by age and arm span which can be used for comparative purposes.

## 5. Conclusions

This study provides the largest data set in an adolescent population specific to the CET to date. These results can be used as a reference point for clinicians and coaches who are using the CET within their assessment. The results revealed that age and arm span are significant predictors of CET and, given this finding, normative data should account for these variables.

## Figures and Tables

**Table 1:** Average CET (cm) distributed by age and gender.

Age	Male		Female		Total	
	n	Mean(SD)	n	Mean(SD)	n	Mean(SD)
<10	2	18.83(0.94)	0	0(0)	2	18.83(0.94)
11	6	20.36(6.90)	9	17.11(6.09)	15	18.4(6.40)
12	33	18.47(8.23)	27	19.05(6.80)	60	18.73(7.56)
13	36	16.34(6.09)	47	18.39(6.82)	83	17.5(6.56)
14	37	16.97(6.42)	29	16.52(8.56)	66	16.78(7.38)
15	66	19.84(8.55)	38	24.0(8.54)	104	21.35(8.74)
16	39	22.21(5.73)	20	23.8(5.68)	59	22.75(5.71)
17	14	24.24(6.79)	10	20.23(7.20)	24	22.57(7.10)
18	1	33.00(0)	2	28.08(14.02)	3	29.72(10.31)
Total	234	19.38(7.53)	182	20.09(7.89)	416	19.69(7.69)

* n = number of participants; SD = standard deviation.

**Table 2:** Standardized values based on age and arm span.

Age (Years)	Arm Span (cm)	Average CET Score (cm)	CET Range (cm)
10–12 (n = 27)	140–154	12.5 ± 3.5	8.4–18.3
	155–169	16.3 ± 7.5	6.3–29.5
	170–184	22.0 ± 6.4	12.8–27.1
13–15 (n = 42)	150–164	15.65 ± 7.7	9.3–31.8
	165–179	19.7 ± 10.4	7.7–40.0
	180–194	24.2 ± 11.1	9.4–39.8
16–18 (n = 36)	160–174	27.2 ± 5.8	17.8–32.8
	175–189	24.0 ± 5.5	14.5–33.5
	190–205	30.3 ± 4.4	26.2–35.0
